# The antioxidant activities of alkalic-extractable polysaccharides from *Coprinus comatus* on alcohol-induced liver injury in mice

**DOI:** 10.1038/s41598-018-30104-6

**Published:** 2018-08-03

**Authors:** Huajie Zhao, Jianjun Zhang, Xinchao Liu, Qihang Yang, Yuhan Dong, Le Jia

**Affiliations:** College of Life Science, Shandong Agricultural University, Taian, 271018 P.R. China

## Abstract

The aim of this work was to provide a preliminary characterization of alkalic-extractable polysaccharides (ALPS) from *Coprinus comatus*, to explore its *in vivo* antioxidant activities and protective effects on alcohol-induced liver injury. ALPS showed strong antioxidant and anti-inflammatory abilities and markedly low serum enzyme activities, hepatic and serum lipid levels, as well as low hepatic lipid peroxidation levels; moreover, ALPS improved the alcohol metabolism system. These results were also confirmed by an analysis of histopathological section observations. ALPS, in both α- and β-configurations, as analysed by fourier-transform infrared (FT-IR) and nuclear magnetic resonance (NMR), was mainly composed of rhamnose (Rha), fucose (Fuc), ribose (Rib), xylose (Xyl), mannose (Man), galactose (Gal) and glucose (Glu) with mass percentages of 0.52%, 1.02%, 0.80%, 0.92%, 3.05%, 2.96% and 90.73%, respectively. These results may offer support for the use of ALPS as a functional food or natural drug source that can prevent and treat alcohol-induced liver injury.

## Introduction

Alcoholic liver disease (ALD) has become a major cause of morbidity and mortality all over the world^1^. The World Health Organization Management of Substance Abuse Team revealed that alcoholism caused 5.9% of all deaths in 2014. ALD, which is induced by excessive alcohol consumption, can lead to a series of clinical symptoms and morphological characteristics of apoptosis such as steatohepatitis, hepatic fibrosis, cirrhosis and hepatocellular carcinoma^2^. Thus, the exploration of effective treatment for ALD and the use of agents for protecting against liver injury induced by alcohol have drawn more and more attention. Meanwhile, increasing evidence has demonstrated that oxidative stress is the primary cause for pathogenesis in ALD^3,4^. During alcohol metabolism, a part of alcohol is metabolized by cytochrome P4502E1 (CYP2E1) accompanied by the production of reactive oxygen species (ROS) such as 1,1-diphenyl-2-picrylhydrazyl (DPPH), superoxide anion and hydroxyl radicals that can damage DNA, RNA, proteins and lipids and thus destroy normal function in cells^5,6^. Currently, there are many synthetic drugs used for ALD treatment, but none are targeted therapies, and they all have side effects^1^. Hence, antioxidants from natural products have garnered great attention for their potential function in prevention and treatment of alcohol-induced liver injury^7^. Mushroom polysaccharides, natural products from the fruiting body, mycelia and spent mushroom substrate, have been verified to have many pharmacological abilities such as antioxidant, hepatoprotective, hypolipidaemic and anti-diabetic activities^8–10^.

*Coprinus comatus*, called Shaggy Ink Cap in China, is a delicious cultivated edible fungus mainly located in China, Korea, Japan and other Asian countries^11^. Many reports have indicated the fruiting body of *C*. *comatus*, composed of polysaccharides, fatty acids, tocopherols, organic acids and phenolic acids lipids, possesses anti-obesity, immunomodulation, hypolipidaemic, antitumour, hypoglycaemic and antibacterial activities^12,13^. Jie *et al*. and Ozalp *et al*. investigated the hypoglycaemic, hypolipidaemic, and antioxidant activities of mycelia selenium-polysaccharides and the hepatoprotective activities of fruiting body polysaccharides from *C*. *comatus*, respectively^14,15^. However, previous research on *C*. *Comatus* mainly focused on biological activity assays of the water-extractable fruiting body and mycelia polysaccharides. There are few publications on the biological activity of the alkalic-extractable polysaccharides (ALPS) from *C*. *comatus*.

Therefore, the objective of this work was to provide a preliminary characterization of ALPS, to investigate the antioxidant *in vitro* and *in vivo* and hepatoprotective activities of ALPS on acute alcohol-induced liver injury in mice.

## Results

### Antioxidant activities *in vitro*

**Figure 1:**
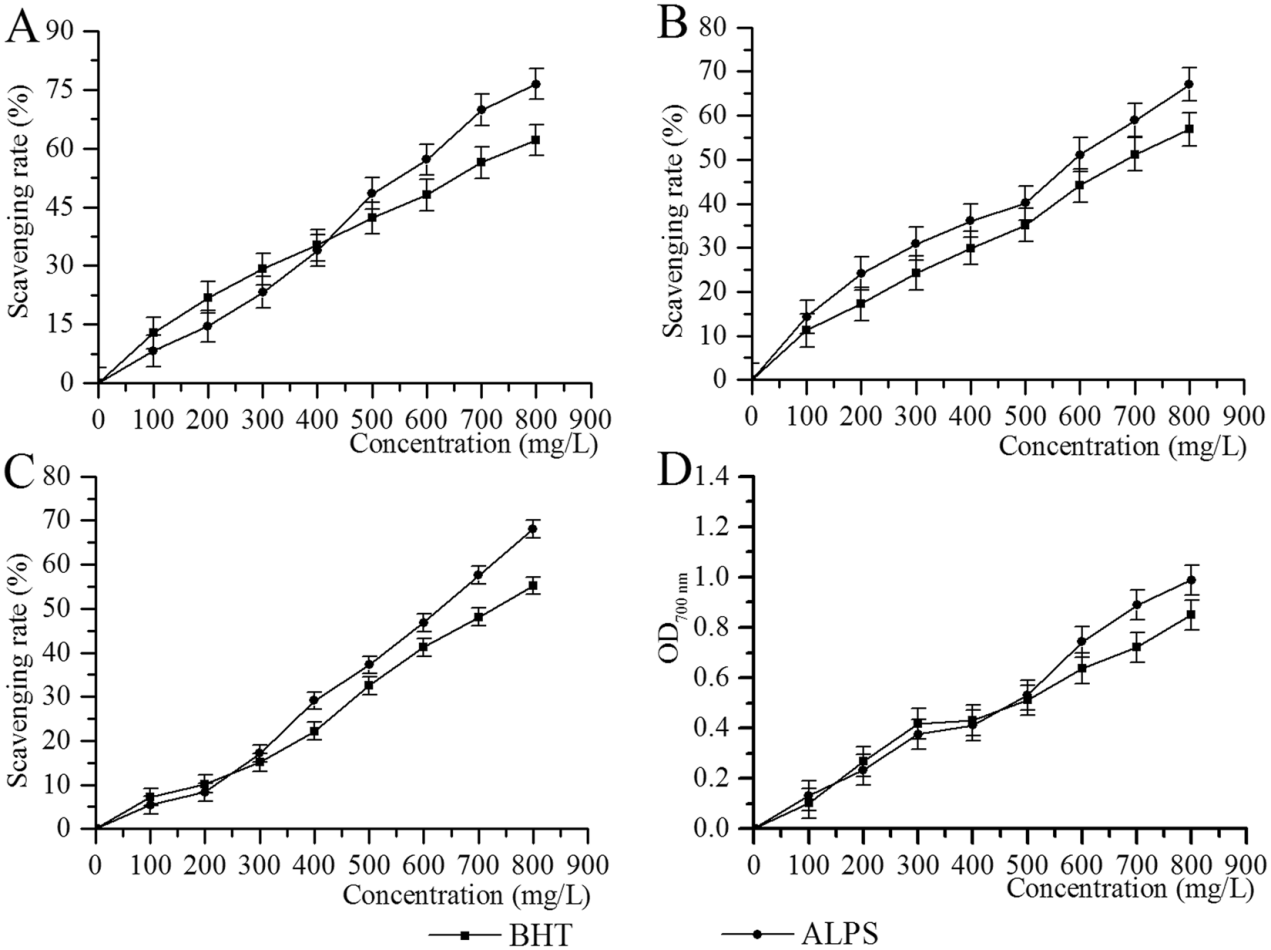
*In vitro* antioxidant activities of ALPS at 0, 100, 200, 300, 400, 500, 600, 700, or 800 mg/L. Scavenging activities towards **(A)** hydrogen peroxide, **(B)** hydroxyl radicals and **(C)** DPPH radicals, and **(D)** reducing power.

ALPS and butylated hydroxytoluene (BHT) exhibited a dose-response inhibition of hydrogen peroxide (Fig. 1A). At a concentration of 800 mg/L, the scavenging activity of ALPS and BHT on hydrogen peroxide was found to be 76.44 ± 3.02 and 62.11 ± 3.45%, respectively. The IC_50_ values of ALPS and BHT were 498.850 ± 2.698 and 603.761 ± 2.781 mg/L, respectively, indicating that ALPS had a potent scavenging effect on hydrogen peroxide.

The scavenging activities of ALPS and BHT on hydroxyl radicals were shown in Fig. 1B and a concentration-dependent effect was observed. Based on the scavenging effects of ALPS (67.07 ± 3.12%) and BHT (56.88 ± 2.34%) at the concentration of 800 mg/L and the IC_50_ values assays of ALPS (560.122 ± 2.748 mg/L) and BHT (725.536 ± 2.861 mg/L), ALPS exhibited very apparent scavenging abilities on hydroxyl radicals.

As displayed in Fig. 1C, the scavenging abilities of ALPS demonstrated a concentration-dependent effect within the concentration range from 0 to 800 mg/L. The scavenging rates of ALPS and BHT towards DPPH radicals reached 68.10 ± 2.84% and 55.21 ± 3.02% at the concentration of 800 mg/L, and the IC_50_ values of ALPS and BHT were 612.812 ± 2.787 and 765.958 ± 2.884 mg/L. These data established that ALPS possessed a strong DPPH radical scavenging ability.

It was observed that reduction potential was increased with the concentration of ALPS and BHT ranging from 0 to 800 mg/L (Fig. 1D). At the concentration of 800 mg/L, the reduction potential of ALPS reached 0.99 ± 0.07, higher than that of BHT at 0.85 ± 0.08, indicating ALPS had a strong reduction ability.

### Acute toxicity study

During the administration of ALPS, mice treated with different oral doses (1000, 2000, 4000 and 6000 mg/kg) did not exhibit any clinical toxicity symptoms or mortality, suggesting that ALPS is a non-toxic product.

### Effects of ALPS on hepatic injury induced by acute alcohol administration

**Figure 2:**
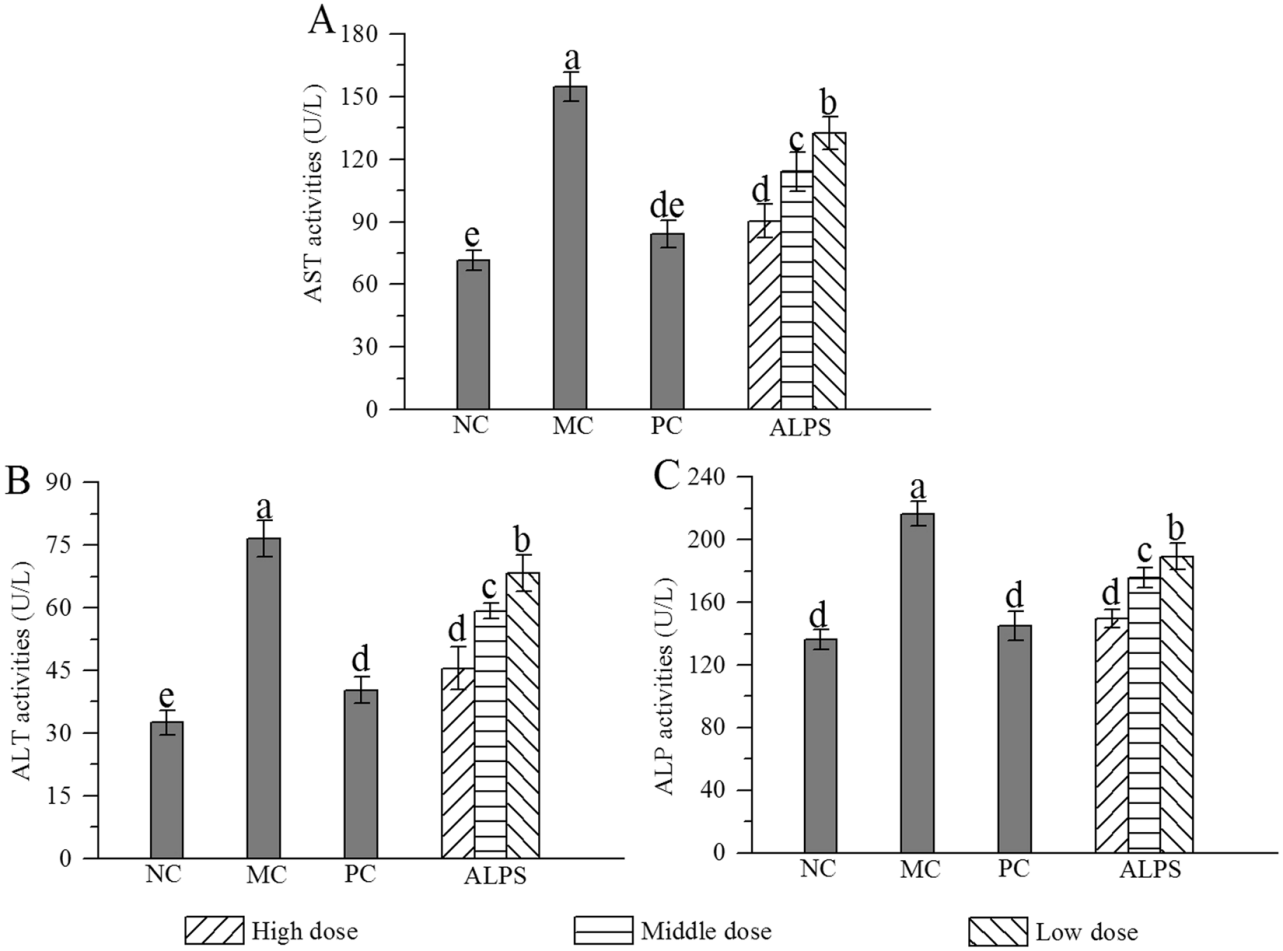
Effects of ALPS on serum enzymes acivities of **(A)** AST, **(B)** ALT and **(C)** ALP in mice treated with ALPS at 200, 400 and 800 mg/kg, bifendatatum at 150 mg/kg, or normal saline for 25 days, and followed by alcohol treatment. NC group had neither ALPS nor alcohol treatments. The values are reported as means ± SD. values not sharing a common superscript letter denote a significant difference (*P* < 0.05).

The aspartate aminotransferase (AST), alanine aminotransferase (ALT) and alkaline phosphatase (ALP) values of serum were 71.4 ± 4.7, 32.5 ± 2.9, 136.27 ± 6.4 U/L in the normal control (NC) group and 154.6 ± 7.1, 76.6 ± 4.3, 216.4 ± 7.9 in the model control (MC) group, respectively (Fig. 2A–C), demonstrating that acute alcohol-induced hepatic damage models were successfully established. However, in mice treated with ALPS (200, 400 or 800 mg/kg) and bifendatatum (150 mg/kg), the elevation of these parameters was inhibited. Briefly, when compared with values from the MC group, the AST, ALT and ALP activities in the high-dose ALPS (H-ALPS) group were decreased by 41.5%, 40.6% and 30.9%, respectively, while these activities were decreased by 45.6%, 47.4% and 33.1% in the positive control (PC) group. These results showed that ALPS provided potential protection against acute alcohol-induced hepatic injury.

**Figure 3:**
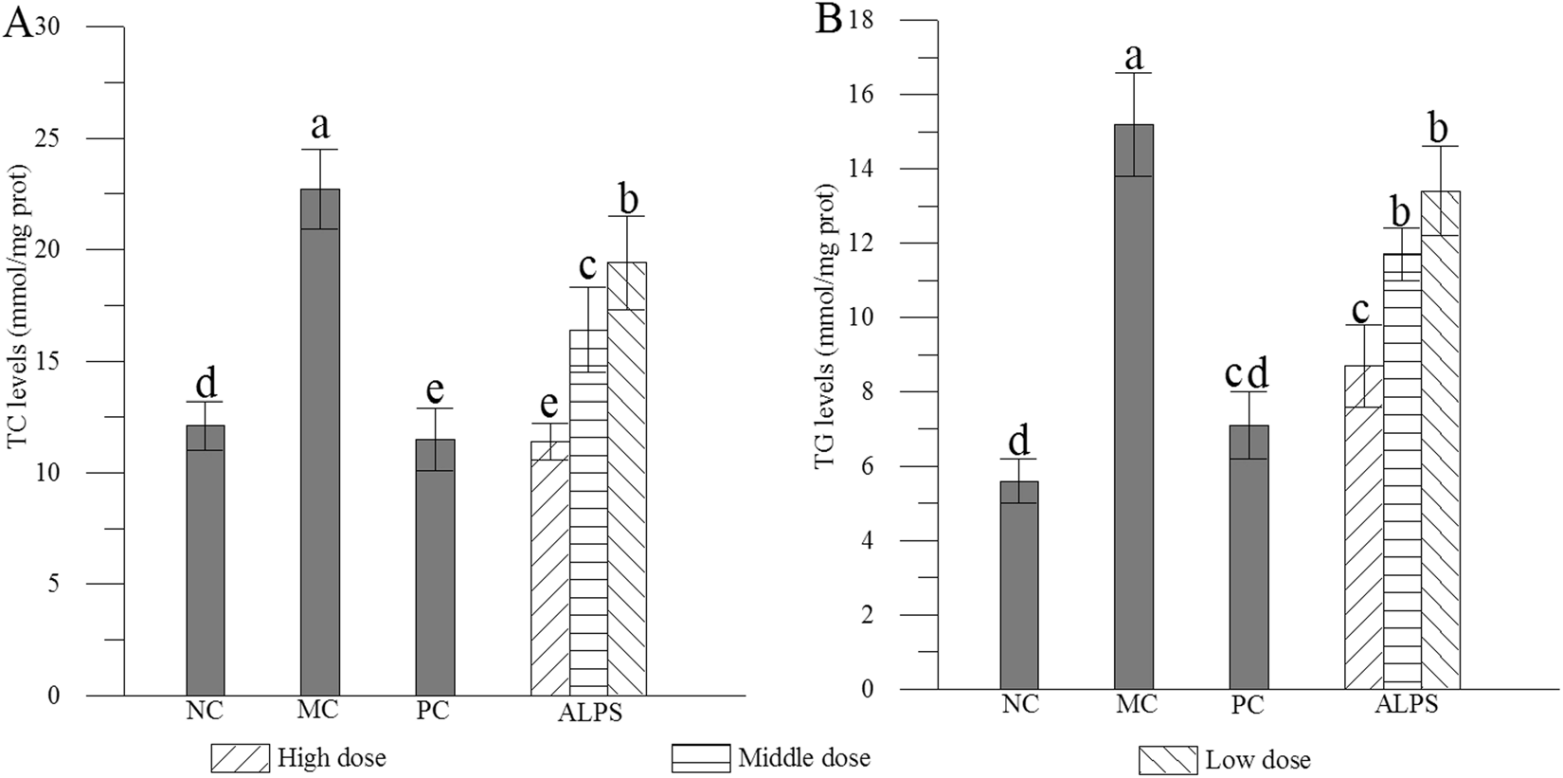
Effects of ALPS on **(A)** TC and **(B)** TG in mice treated with ALPS at 200, 400 and 800 mg/kg, bifendatatum at 150 mg/kg, or normal saline for 25 days, and followed by alcohol treatment. NC group had neither ALPS nor alcohol treatments. The values are reported as means ± SD. values not sharing a common superscript letter denote a significant difference (*P* < 0.05).

Compared with values from the NC group, total cholesterol (TC) and triglyceride (TG) levels of mice in the MC group were markedly increased (Fig. 3). Clearly, pretreatment with ALPS notably improved the abnormal increases of these indexes compared with MC group (*P* < 0.05), suggesting that ALPS may be helpful to repair disorders of hepatic lipid metabolism induced by acute alcohol administration.

**Figure 4:**
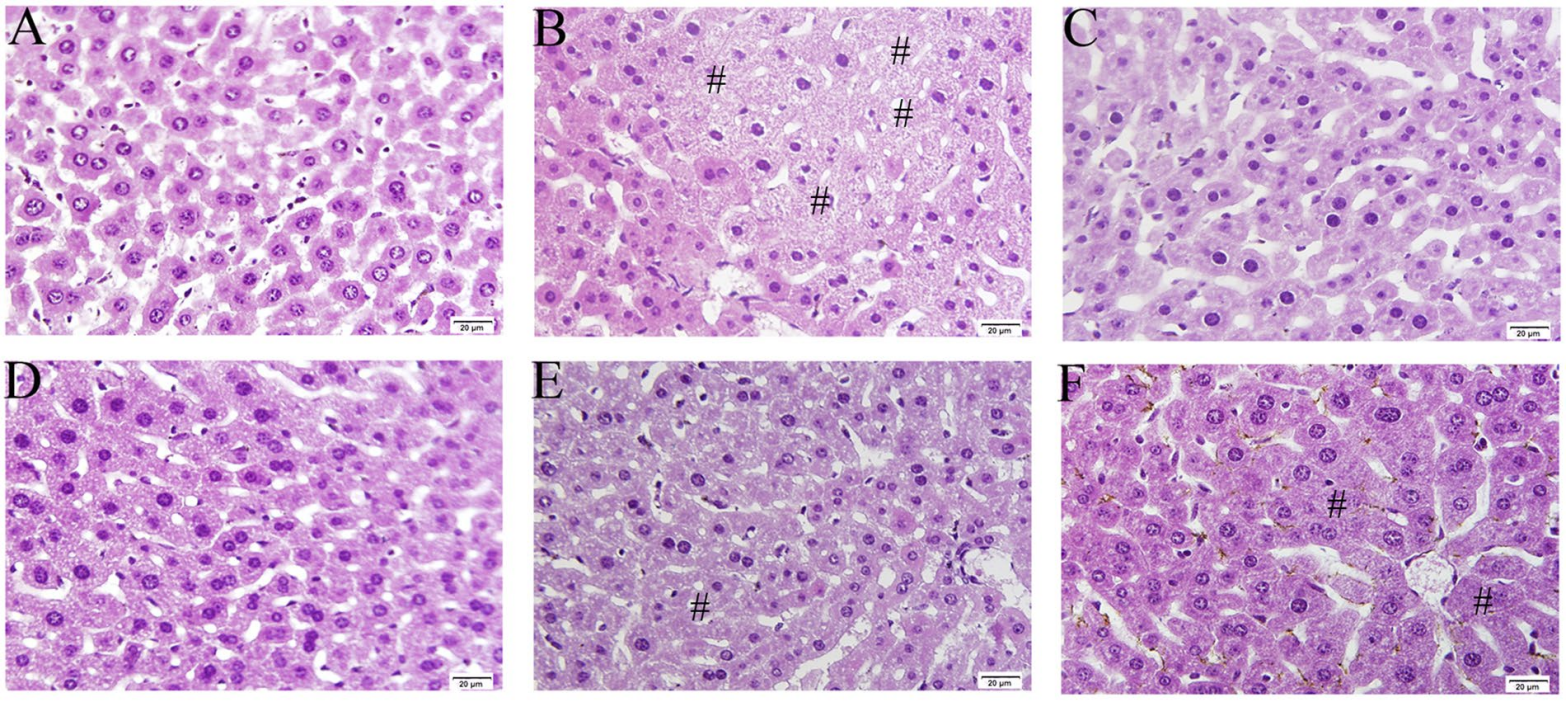
Effects of ALPS on hepatic histological changes stained with H&E in mice treated with ALPS at 200, 400 and 800 mg/kg, bifendatatum at 150 mg/kg, or normal saline for 25 days, and followed by alcohol treatment. NC group had neither ALPS nor alcohol treatments. Magnification: 400×. **(A)** NC group, **(B)** MC group, **(C)** PC group, and **(D**–**F)** groups treated with 800, 400 and 200 mg/kg ALPS. Necrosis (#).

We did not observe distinct histological alterations in mice liver sections in the NC group (Fig. 4A). In contrast, hepatic injury was apparent in the acute alcohol-induced group characterized by loss of cellular boundaries, hepatocyte necrosis, large fat accumulation and nucleus contraction (Fig. 4B). After administration of ALPS, these obvious histological changes were improved to some extent, certifying that ALPS can protect the liver against acute alcohol administration (Fig. 4D–F).

**Figure 5:**
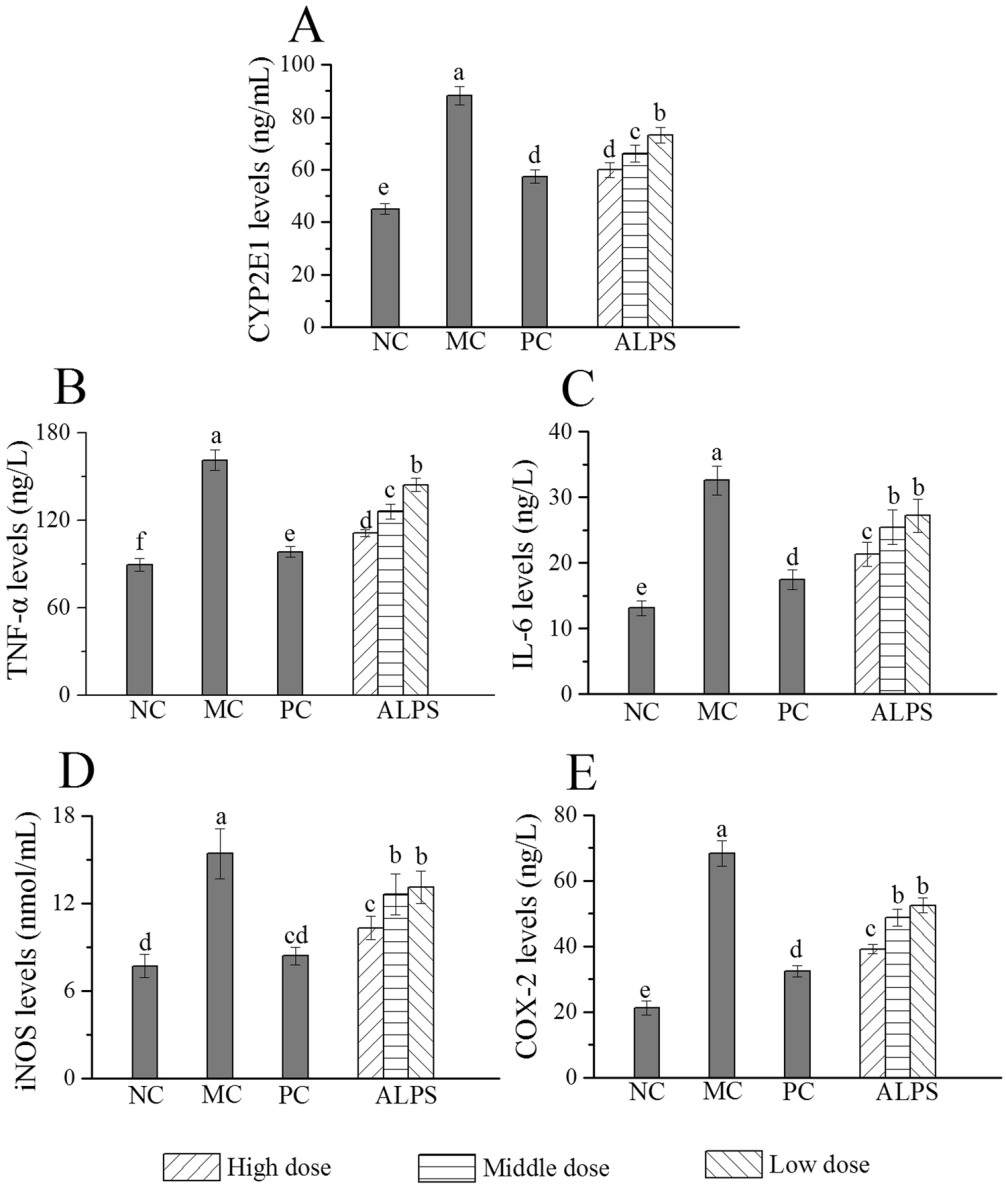
Effects of ALPS on alcohol-metabolizing enzyme, inflammatory cytokines and mediators in mice treated with ALPS at 200, 400 and 800 mg/kg, bifendatatum at 150 mg/kg, or normal saline for 25 days, and followed by alcohol treatment. NC group had neither ALPS nor alcohol treatments. **(A)** CYP2E1, **(B)** TNF-α, **(C)** IL-6, **(D)** iNOS and **(E)** COX-2. The values are reported as means ± SD. values not sharing a common superscript letter denote a significant difference (*P* < 0.05).

Figure 5A displayed the changes of CYP2E1 levels in the different treatment and control groups. The MC group showed significant elevations of CYP2E1 levels in comparison to that of the NC group (*P* < 0.05), illustrating that acute alcohol administration can accelerate the microsome alcohol oxidation system. In PC and ALPS groups CYP2E1 levels were notably reduced following increasing concentrations of ALPS compared with those in the MC group (*P* < 0.05). CYP2E1 levels of mice in the H-ALPS and PC groups were decreased by 32.06% and 34.87%, respectively.

To evaluate the effect of ALPS on the inflammatory response induced by acute alcohol administration, the tumour necrosis factor (TNF-α), interleukin-6 (IL-6), inducible nitric oxide synthase (iNOS) and cyclooxygenase (COX-2) levels in hepatic homogenate was analysed (Fig. 5B–E). Acute alcohol administration increased TNF-α, IL-6, iNOS and COX-2 levels compared to those in the NC group (*P* < 0.05). However, ALPS suppressed the levels of these parameters when compared with those in the MC group (*P* < 0.05).

**Figure 6:**
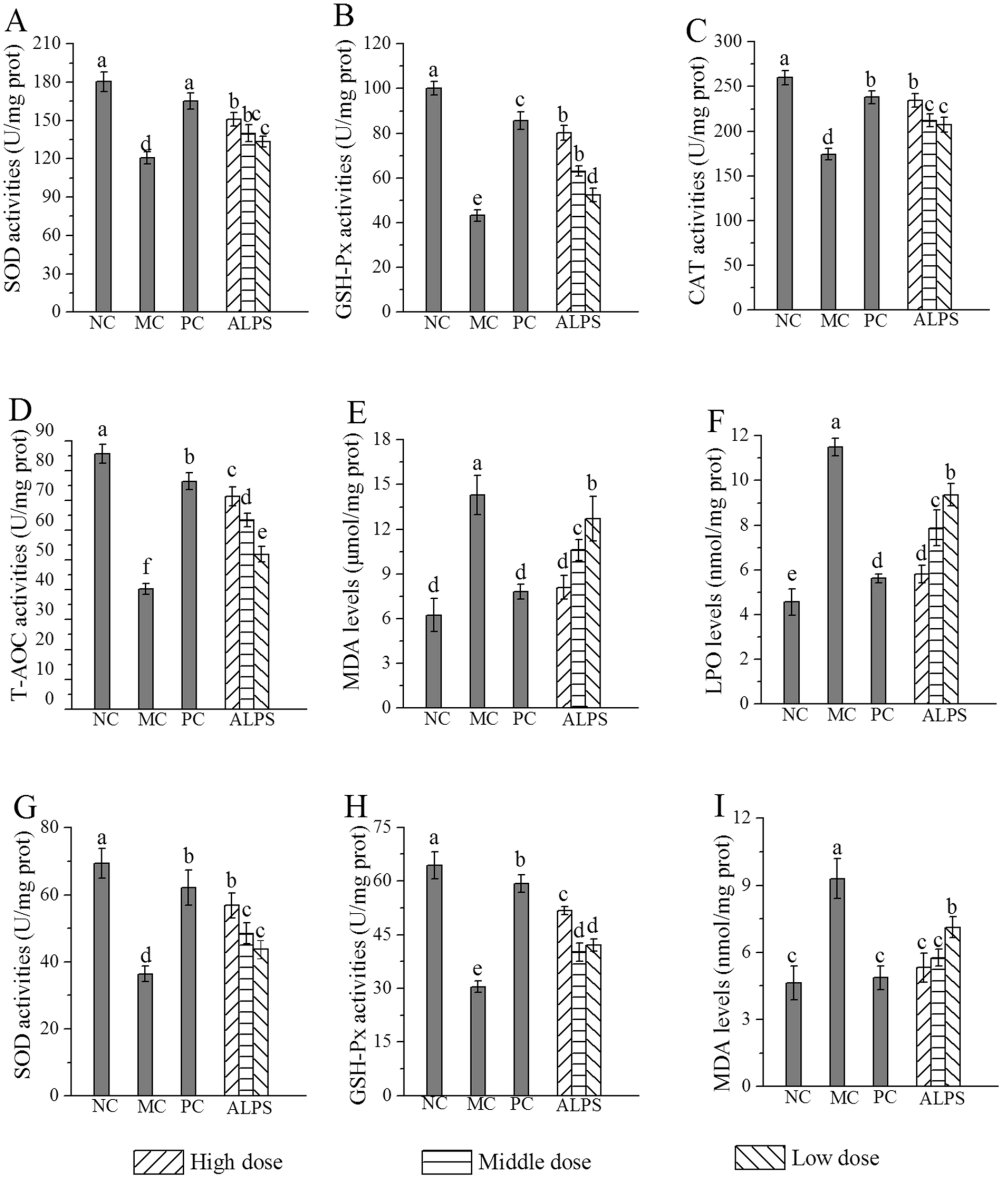
Effects of ALPS on antioxidant enzymes and lipid peroxides in mice treated with ALPS at 200, 400 and 800 mg/kg, bifendatatum at 150 mg/kg, or normal saline for 25 days, and followed by alcohol treatment. NC group had neither ALPS nor alcohol treatments. **(A)** SOD, **(B)** GSH-Px, **(C)** CAT, **(D)** T-AOC, **(E)** MDA, **(F)** LPO in hepatic homogenates, and **(G)** SOD, **(H)** GSH-Px, **(I)** MDA in mitochondria. The values were reported as Means ± SD. Bars with different letters were significantly different (*P* < 0.05).

In comparison to these values in mice from the NC group, the MC group displayed remarkable decreases to the activities of hepatic superoxide dismutase (SOD), glutathione peroxide (GSH-Px), catalase (CAT) and total antioxidant activity (T-AOC); and there were obvious increases in the levels of malondialdehyde (MDA) and lipid peroxidation (LPO) (Fig. 6A–F), affirming that the hepatic antioxidative defence had been damaged by acute alcohol administration. Compared with these values in mice from the MC group, the administration of ALPS significantly elevated the SOD, GSH-Px, CAT and T-AOC activities (from 120.6 ± 4.7, 43.2 ± 2.6, 174.3 ± 6.3 and 40.3 ± 1.9 U/mg prot to 150.8 ± 5.4, 80.2 ± 3.4, 234.6 ± 7.6 and 71.3 ± 3.2 U/mg prot in the H-ALPS group and markedly reduced the MDA and LPO levels (from 14.3 ± 0.9 μmol/mg prot and 11.5 ± 1.3 nmol/mg prot to 8.1 ± 0.8 μmol/mg prot and 5.8 ± 0.4 nmol/mg prot). Furthermore, acute alcohol administration markedly reduced SOD and GSH-Px activities and elevated the MDA level in mitochondria compared with the NC group (*P* < 0.05, Fig. 6G–I). Pretreatment with ALPS inhibited the decrease of SOD and GSH-Px activities and the increase of MDA levels when compared to the MC group. These results showed that ALPS can suppress oxidative stress induced by acute alcohol administration in liver and mitochondria.

### Effects of ALPS on serum lipid levels

**Figure 7:**
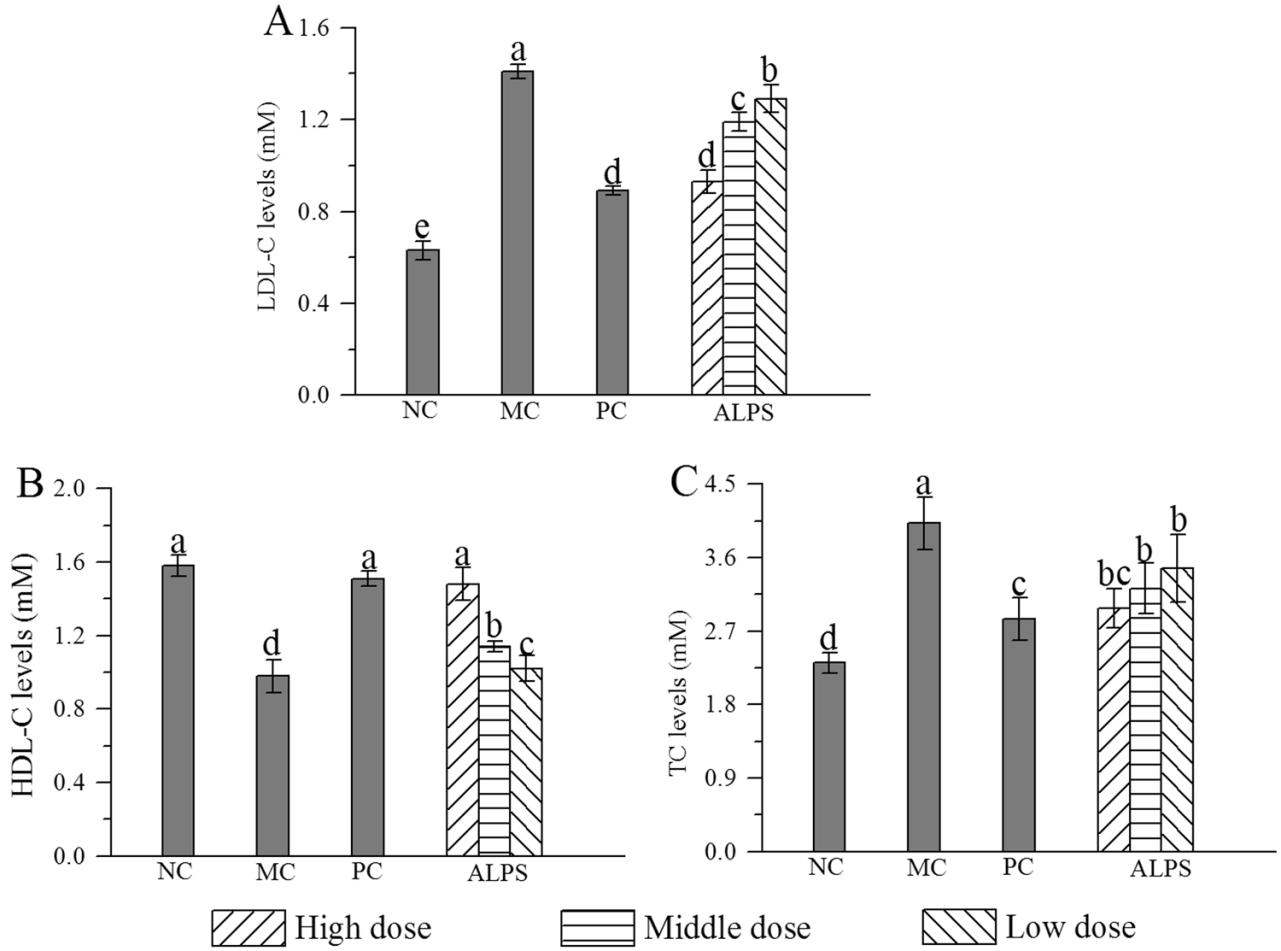
Effects of ALPS on serum lipid profiles in mice treated with ALPS at 200, 400 and 800 mg/kg, bifendatatum at 150 mg/kg, or normal saline for 25 days, and followed by alcohol treatment. NC group had neither ALPS nor alcohol treatments. **(A)** LDL-C, **(B)** HDL-C and **(C)** TC. The values were reported as Means ± SD. Bars with different letters were significantly different (*P* < 0.05).

The low-density lipoprotein cholesterol (LDL-C), high-density lipoprotein cholesterol (HDL-C) and TC levels of the MC group were significantly elevated (with all *P* < 0.05), while HDL-C level was markedly reduced (*P* < 0.05) compared with the NC group (Fig. 7). Interestingly, the abnormal changes in these parameters were partly restored by the supplement of ALPS (with all *P* < 0.05). The serum LDL-C and TC levels reached the minimum in the H-ALPS group, which were 34.0% and 25.9% lower than those of MC group, respectively; whereas the HDL-C level in the H-ALPS group increased by 51.2% compared with the MC group, indicating that ALPS had the potential effect of improving serum lipid metabolism.

### ALPS characterization analysis

**Figure 8:**
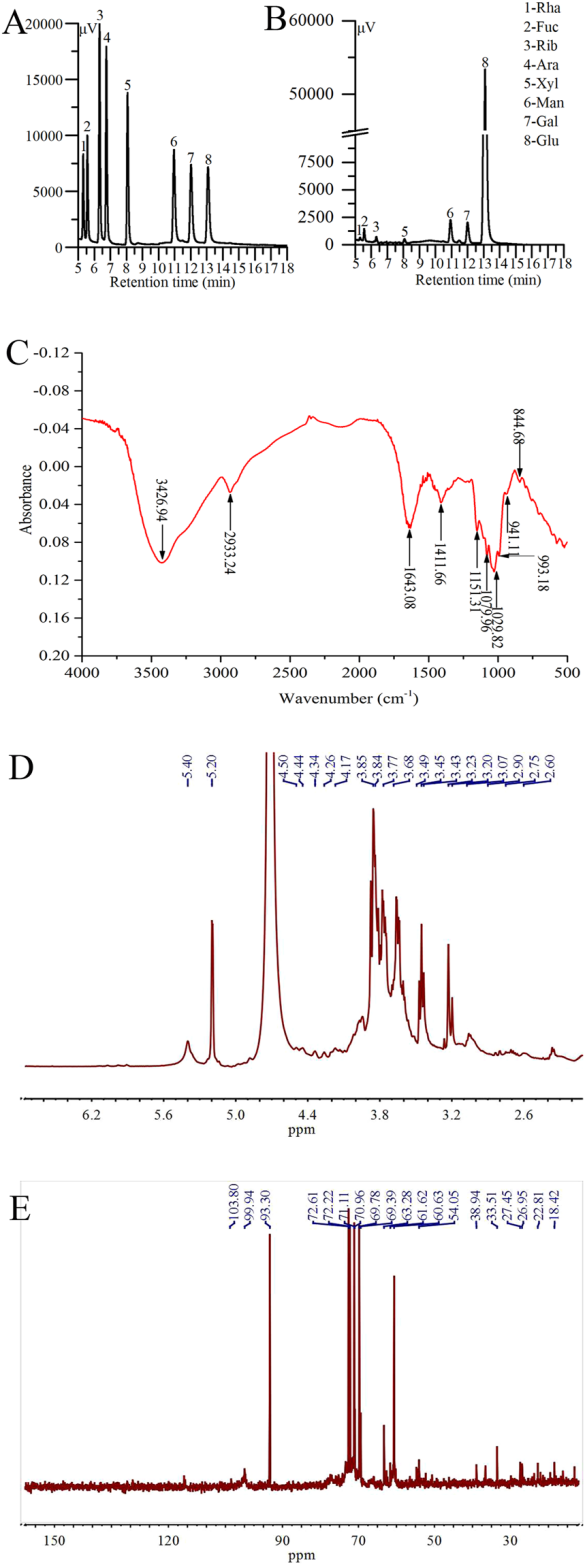
Preliminary characterizations of ALPS. GC chromatograms of **(A)** standard monosaccharides and **(B)** ALPS; **(C)** FT-IR spectrum; **(D)** ^1^H NMR spectrum and **(E)** ^13^C NMR spectrum.

The monosaccharide composition of ALPS was analysed by the retention time of the standard samples’ chromatographic peaks (Fig. 8A). ALPS was composed of Rha, Fuc, Rib, Xyl, Man, Gal and Glu with mass percentages of 0.52%, 1.02%, 0.80%, 0.92%, 3.05%, 2.96% and 90.73%, respectively (Fig. 8B).

The FT-IR spectrum of ALPS showed a wide and strong band at 3426.94 cm^−1^ (derived from the stretching vibrations of -OH), a weak peak at 2933.24 cm^−1^ (ascribed to the stretching and bending vibrations of C-H), peaks at 1643.08 cm^−1^ and 1411.66 cm^−1^ (resulted from the stretching vibrations of -COOH), strong characteristic absorptions at 1200–1000 cm^−1^ (originated from vibrations of C-O-C glycosidic bonds), a weak characteristic absorption peak at 941.11 cm^−1^ (attributed to stretching vibration of α-D-Glucopyranose) and a weak characteristic absorption at 844.68 cm^−1^ (owed to stretching vibration of β-D-Glucopyranose), which showed a typical polysaccharide structure (Fig. 8C).

The ^1^H nuclear magnetic resonance (NMR) spectrum of ALPS is shown in Fig. 8D. ^1^H chemical shifts in the anomeric region were observed from 4 to 6 ppm. The shift signals of β-configuration (4.50, 4.44, 4.34, 4.26 and 4.17 ppm) and α-configuration (5.40 and 5.20 ppm) were displayed^16^. The wide and strong signals from 3.07 to 3.85 ppm, exhibited in the ^1^H NMR spectrum of ALPS, was ascribed to the presence of carbohydrates’ CH_2_-O and CH-O groups^17^. Furthermore, the ^13^C NMR spectrum (Fig. 8E) displayed that signal at 103.80 ppm corresponding to anomeric carbon (C1) signals of glycosides, and signals from 60 to 80 ppm ascribing to C2, C3, C4, C5 and C6 of the glycosidic ring were observed^16^. Meanwhile, signals at 93.40, 99.94 and 103.80 ppm indicated the ALPS had α- and β-configurations^18^.

## Discussion

In our present work, we determined that ALPS can effectively improve dyslipidaemia by negative regulation on LDL-C and TC levels and positive regulation on HDL-C level, alleviate hepatic lipid metabolism disorder by reducing hepatic TC and TG, mitigate oxidative stress *via* enhancing antioxidant enzyme activities and decreasing lipid peroxide products and improve effects from alcohol metabolism disorder by suppressing CYP2E1 activity in mice with acute alcohol-induced hepatic injury. These results manifested that ALPS can be used for improving hepatic injury induced by acute alcohol consumption.

The liver has the highest detoxification ability in mammals and plays a leading role in alcohol metabolism; acute alcohol administration can induce liver damage^19,20^. Clinically, the status of liver damage can be diagnosed *via* assaying the changes in activities or levels of serum enzymes and liver lipids including AST, ALT, ALP, TC and TG^21^. Notably enhanced activities of AST, ALT and ALP, mainly distributed in the liver, shows that liver damage has occurred, which can cause changes of cell membrane permeability thereby causing leakage of hepatic AST, ALT and ALP into blood circulation^22,23^. Moreover, an abnormal increase of hepatic TC and TG levels indicate that a liver lipid metabolism disorder has occurred, which can also diagnose liver damage status. For this work, the administration of different doses of ALPS had obvious inhibiting effects on serum enzyme activities and liver lipid levels, especially high-dose ALPS; these results were also confirmed by liver slice observation. Thus, it was verified that ALPS may contribute to the development of novel treatments against acute alcohol-induced liver damage.

Alcohol-induced oxidative stress plays an important role contributing to the development of ALD^24^. CYP2E1, the foremost contributor to oxidative stress induced by alcohol, belongs to the most important enzyme in the microsome alcohol-oxidation system. It is reported that increased CYP2E1 levels caused by the administration of alcohol can accelerate the overproduction of ROS by a decomposition reaction from alcohol into acetaldehyde^25^. Thus, it is necessary to assay the effects of ALPS on ROS-induced oxidative stress and explore the antioxidant ability of ALPS *in vitro* and *in vivo*. Hydrogen peroxide, a powerful oxidant produced by biological systems, is not very reactive, but it can be toxic to cellular energy generation systems when hydroxyl radicals increase in cells^26,27^. Hydroxyl radicals, a highly reactive species induced by the interaction between iron ions and hydrogen peroxide in biological systems, are responsible for oxidative damage^28^. DPPH, characterized as a stable free radical by electron delocalization effect, can react with an H donor to form DPPH-H (a non-radical state); therefore a DPPH colour change can indicate scavenging ability^21,26^. Reduc potential, described as the ability to interrupt free radical chains and contribute hydrogen atoms, is related to antioxidant ability^29^. Furthermore, antioxidant enzymes (SOD, GSH-Px and CAT) were essential for the endogenous antioxidant defence system to scavenge ROS and maintain the balance of oxidation-reduction in the cell^23,30,31^. SOD, the most antioxidant enzyme in the first line of defence, can disproportionately convert superoxide to hydrogen peroxide that subsequently decomposes into water owing to the presence of CAT^32^. GSH-Px, a selenoenzyme located in the cell cytoplasm, plays a vital role in improving antioxidant defence capability against peroxide radicals and oxygen^33^. T-AOC was applied to evaluate antioxidant defence capacity in the non-enzymatic system of the organs. It was reported that ROS can give rise to LPO, and its end product (MDA) has been widely used to detect LPO and oxidative stress status^34,35^. The present results indicate that ALPS possessed obvious antioxidant capacity *in vitro*, suppressed hepatic CYP2E1, LPO, MDA levels and mitochondrial MDA levels as well as enhanced antioxidant enzyme activities in liver homogenate and mitochondria. Therefore, ALPS had a potentially protective effect on the liver against oxidative stress induced by acute alcohol administration. Meanwhile, Li, Lu, Suo, Nan, & Li have investigated the antioxidant properties of the cap and stipe from *C*. *comatus*^36^. Their maximum scavenging abilities on DPPH and hydroxyl radicals at 5 g/L reached approximately 65% and 61.3%, respectively, lower than 68.1% and 67.07% of ALPS at 0.8 g/L, respectively, indicating ALPS had significantly higher antioxidant activity than the fruiting body extract of *C*. *comatus*.

Some reports have shown that alcohol can activate inflammatory responses and release inflammatory cytokines, such as TNF-α and IL-6, which accelerate ALD^37^. TNF-α can expedite neutrophil migration, and they augment the production of proteolytic enzymes and ROS thereby accelerating hepatic injury^38^. IL-6 plays an important role in promoting hepatic damage and its abnormal increase induces inflammatory diseases and cancers^39^. Meanwhile, alcohol can also induce the overexpression of inflammatory mediators such as iNOS and COX-2^37^. iNOS and COX-2 can cause the production of down-stream factors that play pivotal roles in the pathogenesis of acute and chronic liver inflammation^40^. Experimental investigations have shown that acute alcohol administration markedly enhances the levels of TNF-α, IL-6, iNOS and COX-2. Interestingly, these inflammatory cytokines and mediators were significantly inhibited by treatment with ALPS, indicating that ALPS has a potential anti-inflammatory ability.

Many reports have shown that oxidative stress can increase the incidence of hyperlipidaemia, diagnosed by assaying the levels of LDL-C, HDL-C and TC^41^. Previous studies reported that HDL (a “beneficial” lipoprotein for human health) can transport superfluous TC from peripheral tissues/cells to the liver for katabolism by blood circulation thereby reducing the risk of TC^42^. However, LDL-C, as the main carrier, can inhibit the transportation of TC so that the combination of LDL-C and TC is gathered in blood vessel walls causing increased plasma lipids^43^. Thus, low levels of HDL-C and high levels of LDL-C and TC are harmful to human health. In this work, the TC and LDL-C levels were increased, and HDL-C level was reduced in MC mice groups; as such, they were improved by treatment with ALPS.

A GC analysis for ALPS showed seven different monosaccharides and a dominance of glucose, followed by Man, Gal, Fuc, Xyl, Rib and Rha. Compared with other reports, the monosaccharide composition of ALPS was different from the enzymatic-extractable mycelia selenium polysaccharide from *Agrocybe cylindracea*^44^, the exopolysaccharide from *Termitomyces albuminosus*^21^ and the fruiting body polysaccharide from *Catathelasma ventricosum*^45^. Furthermore, ALPS displayed the typical absorption of polysaccharides with α- and β-configuration pyranose upon FT-IR spectrum analysis; this result was also confirmed by NMR spectrum analysis. However, polysaccharides configurations from *Grifola frondosa* having α-configurations^17^ and *Polyporus albicans* having β-configurations^46^ were different from ALPS configurations. These differences might be attributed to different strains, culture methods, and extraction conditions.

## Materials and Methods

### Materials, chemicals and animals

The fruiting body of *C*. *comatus* was provided by Beijing Engineering Research Center for Edible Mushrooms (Beijing, China). The reagent kits for analysing SOD, GSH-Px, CAT, T-AOC, MDA, LPO, CYP2E1, TC and TG were purchased from Nanjing Jiancheng Bioengineering Co. Ltd., (Nanjing, China). The ELISA kits of CYP2E1, TNF-α, IL-6, iNOS and COX-2 were obtained from Jiangsu Meibiao Biological Technology Co. Ltd., (Jiangsu, China). DPPH and standard monosaccharides (Rha, Ara, Xyl, Man, Gal, Glu, Rib and Fuc) were purchased from Sigma Chemicals Co. Ltd., (St. Louis, USA). All other chemicals of analytical grade used in this experiment were obtained from local chemical suppliers. The Kunming strain mice (males, 20 ± 2 g) were purchased from Taibang Biological Products, Inc. (Tai’an, China).

### Isolation of ALPS

The fresh fruiting body of *C*. *comatus* was dried and ground into a powder that was mixed with suitable volumes of sodium hydroxide solution (1 mol/L) at 80 °C for 3 hours. After centrifugation at 3000 rpm, the supernatant liquor was gathered, and the resulting precipitate was handled once more twice as mentioned above. The collected supernatant liquor was concentrated at 50 °C, neutralized by HCl solution (1 mol/L), precipitated with three volumes of ethanol (95%, v/v), shaken well, and kept at 4 °C overnight. After centrifugation (3000 rpm), the precipitate was deproteinated by the Sevage method^47^, dialyzed and lyophilized for further experiment. The polysaccharide content was determined using the phenol-sulphuric acid colourimetric method^48^.

### Antioxidant activities *in vitro*

The scavenging activity of ALPS on hydrogen peroxide was investigated as previously reported^49^. A reaction mixture including phosphate buffer (2 mL, 0.1 mol/L, pH 7.4), H_2_O_2_ solution (0.4 mL, 0.3%, v/v) and ALPS (1 mL, 0–800 mg/L) was determined at 230 nm after 15 minutes by spectrophotometer using deionized water as a blank control and BHT as a positive standard. The scavenging rate of ALPS was evaluated using the following formula

1 $${\rm{Scavenging}}\,{\rm{rate}}\,( \% )=[({{\rm{A}}}_{0}-{{\rm{A}}}_{1})/{{\rm{A}}}_{0}]\times 100$$

where A_0_ is the absorbance of the blank control and A_1_ is the absorbance in the presence of ALPS or BHT.

The scavenging ability of ALPS on hydroxyl radicals was evaluated reported^26^. The mixture, composed of KH_2_PO_4_ (10 mmol/L, pH 7.4), deoxyribose (25 mmol/L), ferric chloride (10 mmol/L), ascorbic acid (100 mmol/L), H_2_O_2_ (28 mmol/L) and ALPS (1 mL, 0–800 mg/L), was incubated at 37 °C for 1 hour. Thiobarbituric acid (1 mL, 1%, w/v) and trichloroacetic acid (1 mL, 3%, w/v) were added to the above mixture. After incubating at 100 °C for 20 minutes, the absorbance of the mixture was evaluated at 532 nm accompanied by a blank control containing all the above reagents and deionized water instead of ALPS, and by BHT as a positive control. The scavenging rate of ALPS on hydroxyl radicals was expressed as formula 1.

The scavenging activity of ALPS on DPPH radicals was evaluated using the previously described method^50^. The ALPS (1 mL, 0–800 mg/L) and DPPH solution (1 mL, 0.004%, w/v) in methanol were mixed, shaken up and placed in a dark at room temperature for 30 minutes. The absorbance of the mixture was obtained at 517 nm using deionized water as a blank control and BHT as a positive standard. The scavenging rate of ALPS on DPPH radicals was calculated from the formula 1.

The reduction potential of ALPS was measured according to the method reported by Oyaizu^51^. A reaction mixture containing ALPS (1 mL, 0–800 mg/L), phosphate buffer (2.5 mL, 0.2 mol/L, pH 6.6) and potassium hexacyanoferrate II (2.5 mL, 1%, w/v) was kept at 50 °C for 20 minutes. TCA (2.5 mL, 10%, w/v) was added to the mixture and centrifuged at 3000 rpm for 10 minutes. Subsequently, deionized water (2.5 mL) and FeCl_3_ (0.5 mL, 0.1%, w/v) were merged with the supernatant (2.5 mL). The absorbance was recorded at 700 nm using deionized water as a blank control and BHT as a known standard.

The IC_50_ values (μg/mL) of scavenging hydrogen peroxide, hydroxyl radicals and DPPH radicals were defined as the effective concentrations of the sample at which the radicals were inhibited by 50%.

### Acute toxicity study

The acute toxicity of ALPS was evaluated using the methods reported by Dib *et al*.^52^. Twenty Kunming mice (male, 20 ± 2 g) were randomly divided into five groups (five mice per group). The experimental group of mice were treated with different doses of ALPS (1000, 2000, 4000 and 6000 mg/kg) and the control group mice received isopycnic normal saline. The behavioural changes or mortality of all mice were observed for 48 hours.

### Animal experiments

The Kunming strain mice were housed in a standardized animal room with a controlled temperature of 23 ± 2 °C, relative humidity of 50 ± 5% and a cycle of 12/12 hours light/dark and allowed free access to food and water. The experiments were performed according to procedures approved by the Institutional Animal Care and Use Committee of Shandong Agricultural University in accordance with the Animals (Scientific Procedures) Act of 1986 (amended 2013).

The 7-day-old domesticated mice were divided into six groups (ten mice per group) including an NC group, MC group, PC group and three ALPS-treated groups. During experiment procedures, mice in the NC and MC groups received normal saline and mice in the PC group were administered bifendatatum (150 mg/kg), whereas mice in ALPS-treated groups were administered ALPS at doses of 200, 400 and 800 mg/kg. ALPS, normal saline and bifendatatum were fed to mice once daily by filling the stomach with a syringe for 25 consecutive days.

On the twenty-sixth day, all mice except the NC group were injected intraperitoneally with alcohol (50%, v/v, 12 mL/kg) every 12 hours three times then sacrificed *via* anaesthesia by diethyl ether. Blood was obtained from the retrobulbar vein, centrifuged (5000 rpm) for 10 minutes at 4 °C to obtain the serum. The AST, ALT and ALP activities, as well as LDL-C, HDL-C and TC levels, were measured by an automatic biochemical analyser (ACE, USA). The livers were rapidly excised, weighed and homogenized (1:9, w/v) in phosphate buffer (0.2 mol/L, pH 7.4, 4 °C). After centrifugation at 3000 rpm for 10 minutes, the supernatant was collected for the evaluation of SOD, GSH-Px, CAT and T-AOC as well as the levels of CYP2E1, MDA, LPO, TC, TG, TNF-α, IL-6, iNOS and COX-2 using commercial reagent kits according to the manufacturer’s instructions. Liver mitochondria were separated using a previously reported method with slight modification^53^. Briefly, liver tissues were homogenized (1:9, w/v) in cold normal saline. After being centrifuged (3000 rpm, 10 minutes, 4 °C), the collected supernatant was centrifuged (10,000 rpm, 15 minutes, 4 °C) again to cause precipitation. Then, the precipitate was washed twice with cold normal saline and resuspended with normal saline to obtain liver mitochondria suspension for evaluating SOD and GSH-Px activities and MDA levels using commercial reagent kits per their instructions.

The collected liver samples were fixed in 4% formalin for more than one day and embedded in paraffin. The slices (4–5 µm thickness), prepared using a microtome and stained with haematoxylin-eosin (H&E), were observed and photographed under a microscope (×400 magnifications) to reveal the hepatic histopathological changes.

### ALPS characterization analysis

#### Monosaccharide composition analysis

The monosaccharide composition of ALPS was evaluated by gas chromatography (GC-2010, Shimadzu, Japan) equipped with an Rtx-1 capillary column (30 mm × 0.25 mm × 0.25 μm) based on our previously described method^21^. Identification of monosaccharide components was accomplished by comparison with standard monosaccharides of Rha, Ara, Xyl, Man, Gal, Glu, Rib and Fuc.

#### FT-IR spectroscopy analysis

The FT-IR spectrum analysis was recorded on a 6700 Nicolet Fourier transform-infrared spectrophotometer (Thermo Fisher Scientific Co., USA) within the scope of 4000–500 cm^−1^, using the potassium bromide disc method to prepare the specimen.

#### ^1^H and ^13^C NMR spectroscopy analysis

^1^H and ^13^C NMR spectrums were recorded using a Bruker AV-300 spectrometer operating at 300 MHz at 25 °C, and the sample was dissolved in deuterated water.

### Statistical analysis

SPSS software was applied for the statistical evaluation. All data were expressed as the means ± SD (standard deviations). Statistical analyses were carried out by one-way ANOVA using Duncan’s multiple range tests. Differences between experimental groups were considered statistically significant if *P* < 0.05.

## Conclusions

FT-IR and NMR spectrum, used for structural analysis, manifested that ALPS had the typical absorption of polysaccharides with α- and β-configuration pyranose. ALPS was comprised of seven kinds of monosaccharide, including Rha, Fuc, Rib, Xyl, Man, Gal and Glu by GC analysis. ALPS also exhibited pharmacological *in vitro* and *in vivo* antioxidant and hepatoprotective effects on liver injury induced by acute alcohol administration in mice, indicating that ALPS may be a potential natural source and functional food for the treatment and prevention of acute-alcohol-induced liver injury.

## Electronic supplementary material

Supplementary Information
